# An unusual presentation of conjunctival squamous cell carcinoma mistaken for a stye

**DOI:** 10.1016/j.jdcr.2024.05.034

**Published:** 2024-06-08

**Authors:** Eva Shelton, Andrew Swanson, Annika Weinhammer

**Affiliations:** a Department of Dermatology, University of Wisconsin, Madison, Wisconsin; b University of Wisconsin School of Medicine and Public Health, Madison, Wisconsin

**Keywords:** Conjunctival squamous cell carcinoma (SCC), exenteration, Mohs surgery, ocular surface squamous neoplasia (OSSN)

## Introduction

Squamous cell carcinoma (SCC) of the conjunctiva, within the spectrum of ocular surface squamous neoplasia, is a rare yet clinically significant malignancy. With a reported incidence of 0.01-1.38 persons per 100,000 population per year,^1^ it presents unique diagnostic challenges. These tumors typically originate in sun-damaged conjunctiva at the limbus and can be associated with ultraviolet radiation, immunodeficiency, and human papillomavirus infection.^1^, ^2^, ^3^

The clinical manifestations of conjunctival SCC can range from early-stage irritation and redness to advanced symptoms such as a raised eye mass and leucoplakia.^3^ The nonspecific nature of these early symptoms can lead to misdiagnosis, posing a considerable diagnostic challenge for physicians. There is a spectrum of reported treatment modalities for conjunctival SCC including both topical and surgical interventions but there is limited supporting data given the rarity of the condition.^4^

In this report, we present an unusual and challenging case of conjunctival SCC in a 62-year-old woman whose condition was initially mistaken for a stye and ultimately necessitated exenteration for margin control. Through this case, we aim to elucidate the complexities of diagnosing conjunctival SCC and identify key hallmarks for an accurate diagnosis.

## Case report

The patient is a 62-year-old woman who initially sought medical attention for irritation of the left lower eyelid, accompanied by worsening swelling, redness, itch, pain, and a foreign body sensation. The initial presumption of chalazion and meibomianitis guided supportive treatments including hot compresses, lid wipes, and topical and oral antibiotics. After 5 months of topical and oral antibiotics without resolution of symptoms despite the multitude of treatments initiated, she was referred to an ophthalmologist for further management. A biopsy from the left upper eyelid was performed which revealed invasive SCC. Given the ill-defined cutaneous changes on both her upper and lower eyelid, 3 additional biopsies from the left lower eyelid were performed which showed severe dysplasia/SCC in situ. Recognizing the high-risk nature of the malignancy, she was referred for Mohs surgery.

**Fig 1:**
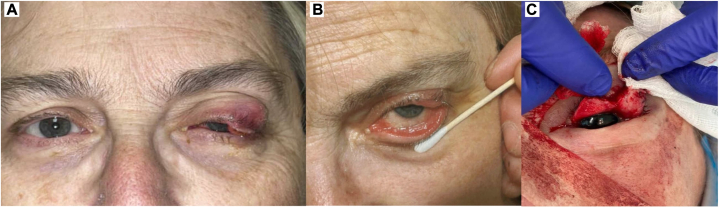
**A-C,** Diffuse erythema and swelling of the upper and lower eyelid and verrucous appearance of the conjunctiva on initial presentation to dermatology.

**Fig 2:**
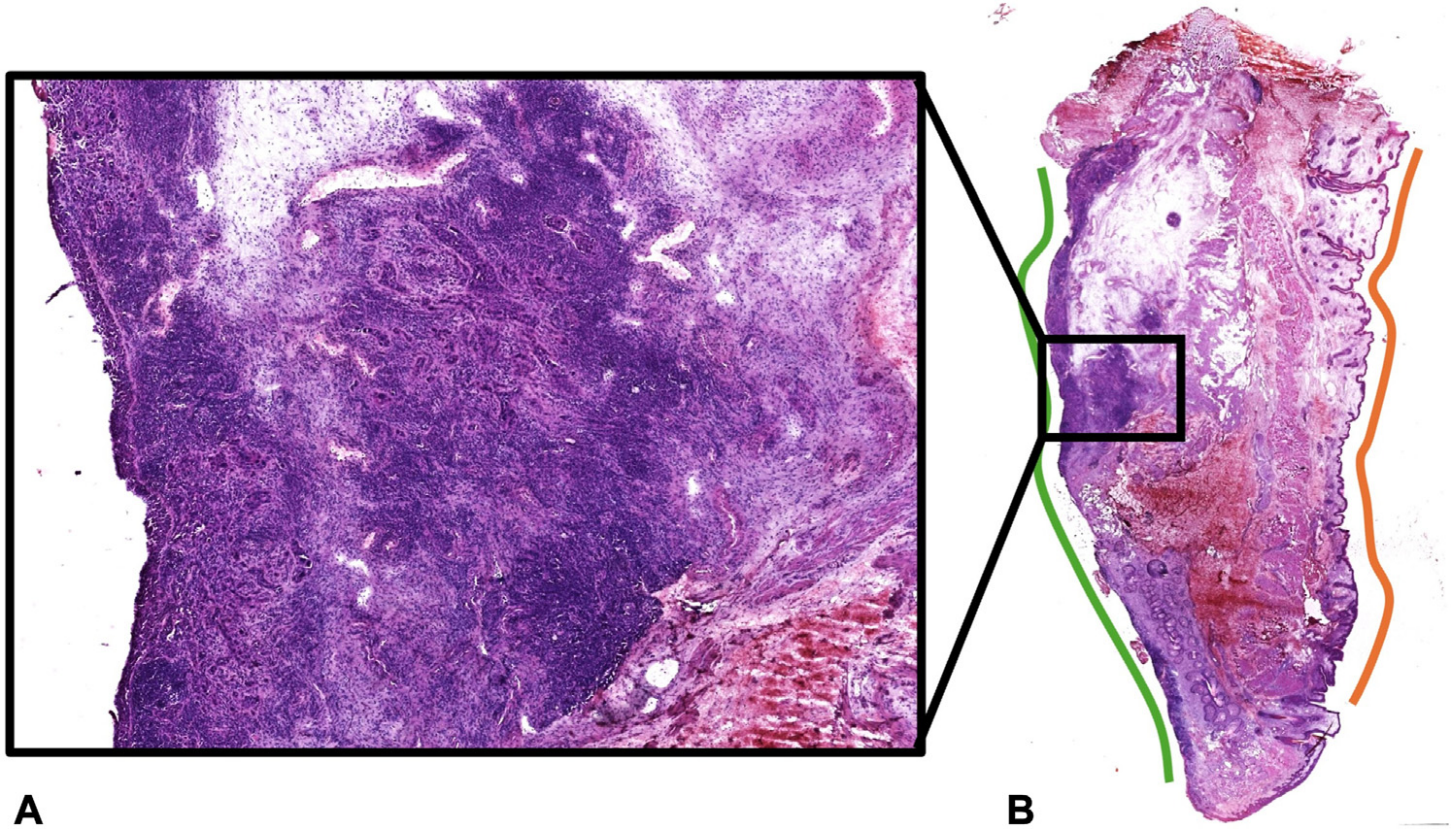
**A** and **B,** Histological examination of the first stage of Mohs surgery. **A,** Reveals islands of large, atypical epithelioid cells with increased nucleus to cytoplasm ratio within the subconjunctival dermis and within the full-thickness epidermis and full-thickness conjunctiva. **B,** Highlights residual squamous cell carcinoma diffusely positive along most of the conjunctival surface (*green*) with minimal involvement of the epidermal surface (*orange*).

**Fig 3:**
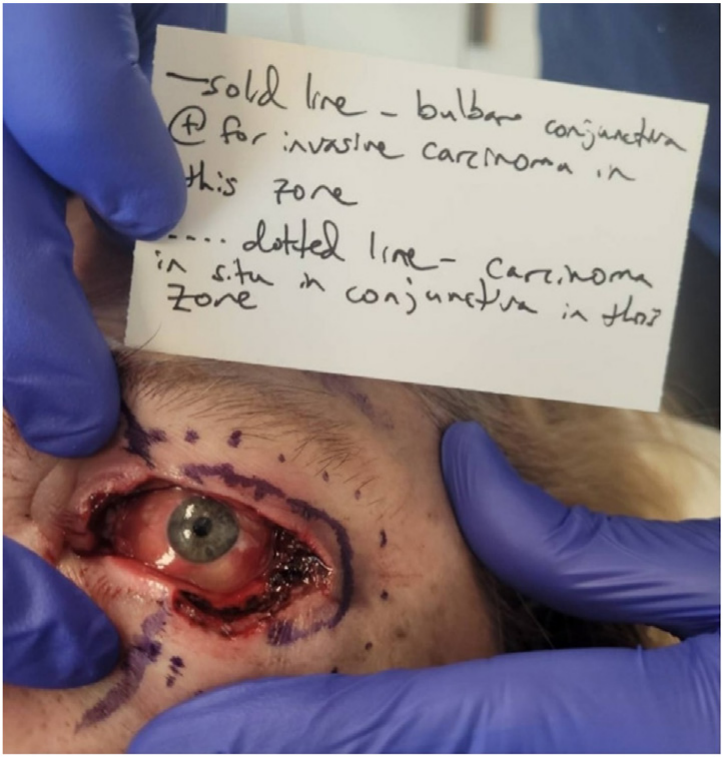
Photo taken at the end of Mohs surgery showing invasive carcinoma and carcinoma in situ tracking onto the bulbar conjunctiva beneath the eye shield that could not be excised safely during Mohs surgery.

Upon presentation to Mohs surgery, initial clinical appearance was notable for diffuse erythema and swelling of the upper and lower eyelid and a diffusely verrucous appearance of the conjunctiva (Fig 1). Histological examination of the first stage revealed islands of large, atypical epithelioid cells with increased nucleus-to-cytoplasm ratio within the subconjunctival dermis (moderate and poorly differentiated SCC) and within the full-thickness epidermis and full-thickness conjunctiva (Fig 2). In all stages, involvement of epidermis was minimal, and the majority of carcinoma was present on the conjunctival surface, which was diffusely positive in nearly all stages (Fig 2, *B*). After the second stage, it was noted that invasive carcinoma was tracking onto the bulbar conjunctiva beneath the eye shield and could not be excised safely in this setting. Two more additional stages were taken to remove any safely accessible tumor. Next, we discussed with the patient our recommendation to discontinue Mohs surgery despite the remaining invasive carcinoma and carcinoma in situ within the conjunctiva (Fig 3).

Ophthalmology was promptly contacted given our strong clinical suspicion for a primary conjunctival SCC, evidenced by the extensive conjunctival involvement with minimal cutaneous eyelid involvement. Jointly with ophthalmology, we thoroughly discussed treatment options with patient, including exenteration for tumor control and less effective options such as 5-fluorouracil eyedrops and mitomycin C. The patient opted for exenteration, citing desire to maximize her odds of survival and not delay definitive treatment. Exenteration was performed 3 days after Mohs surgery which successfully removed the entire tumor, encompassing the inferior palpebral conjunctiva, superior and inferior bulbar conjunctiva. Notably, she also had dysplasia in the Meibomian ducts, although she did not have perineural or vascular invasion. Post-operatively, the patient recovered well without complications.

## Discussion

The presented case brings attention to the intricate diagnostic challenges associated with conjunctival SCC and the potential repercussions of misdiagnosis. Despite its infrequent occurrence, the aggressive nature of these tumors mandates a prompt and precise diagnosis for effective management. In this case, the patient's early symptoms led to the initial misdiagnosis of a stye or cellulitis involving the eyelid. The elusive symptoms of conjunctival SCC, combined with its rarity, posed a considerable diagnostic challenge which was not identified until treatment. Ultimately, the meticulous evaluation during Mohs surgery revealed extensive involvement of both the palpebral and bulbar conjunctiva, which made it evident that the origin of the tumor was from the conjunctiva. This difficult diagnosis highlights the intricacies involved in the management of conjunctival SCC and emphasizes the need to consider it in the differential diagnosis. Since its early symptoms overlap with other common eyelid conditions, consideration of conjunctival SCC is particularly important when standard treatments for presumed eyelid conditions fail to produce the expected improvement. This approach may help clinicians identify the diagnosis earlier.

Treatment options include cryotherapy, 5-fluorouracil, mitomycin C, radiotherapy, peripheral and deep en face margin assessment, and exenteration.^4^^,^^5^ Mohs surgery was initially pursued for this patient which allowed for thorough evaluation and partial removal, but it became evident that the lesion extended beyond the confines of Mohs surgery and required exenteration for full removal. Coordinated ophthalmologic care was imperative and detailed photos of each stage was prudent to keep the clinician informed and prepared. The decision to proceed with exenteration for this patient, given the extensive nature of her disease, underscores the imperative to tailor treatment to the individual, taking into consideration the disease extent, associated risks, and patient preferences.

In conclusion, this case highlights the intricacies in diagnosing conjunctival SCC, particularly when initially misconstrued as common eyelid conditions such as stye and cellulitis, and later as eyelid SCC. Timely recognition, thorough evaluation, and collaborative efforts among different specialties are imperative for optimal management and favorable outcomes in conjunctival SCC cases. Further research and heightened awareness within the medical community are indispensable to advance the early detection and treatment of this rare yet potentially devastating ocular malignancy, considering a nuanced spectrum of treatment options tailored to individual cases.

## Conflicts of interest

None disclosed.
